# Potential role of bacteria packaging by protozoa in the persistence and transmission of pathogenic bacteria

**DOI:** 10.3389/fmicb.2014.00240

**Published:** 2014-05-21

**Authors:** Alix M. Denoncourt, Valérie E. Paquet, Steve J. Charette

**Affiliations:** ^1^Institut de Biologie Intégrative et des Systèmes, Université LavalQuebec City, QC, Canada; ^2^Centre de Recherche de l'Institut Universitaire de Cardiologie et de Pneumologie de QuébecQuebec City, QC, Canada; ^3^Département de Biochimie, de Microbiologie et de Bio-Informatique, Faculté des Sciences et de Génie, Université LavalQuebec City, QC, Canada

**Keywords:** protozoa, multilamellar body, amoeba, bacteria packaging, *Legionella pneumophila*, mycobacteria, persistence, transmission

## Abstract

Many pathogenic bacteria live in close association with protozoa. These unicellular eukaryotic microorganisms are ubiquitous in various environments. A number of protozoa such as amoebae and ciliates ingest pathogenic bacteria, package them usually in membrane structures, and then release them into the environment. Packaged bacteria are more resistant to various stresses and are more apt to survive than free bacteria. New evidence indicates that protozoa and not bacteria control the packaging process. It is possible that packaging is more common than suspected and may play a major role in the persistence and transmission of pathogenic bacteria. To confirm the role of packaging in the propagation of infections, it is vital that the molecular mechanisms governing the packaging of bacteria by protozoa be identified as well as elements related to the ecology of this process in order to determine whether packaging acts as a Trojan Horse.

## Introduction

The risk of the resurgence of bacterial infections caused by the decreasing effectiveness of antibiotics and the increasing number of people with weakened immune systems (cancer, AIDS, aging, etc.) require our attention (Croft et al., 2007). In this context, it is crucial that we improve our understanding of the behavior of pathogenic bacteria in various environments and their transmission in order to develop effective countermeasures. Since many protozoa interact with pathogenic bacteria in diverse environments, understanding the behavior of pathogens includes elucidating their relationships with protozoa.

Protozoa are unicellular eukaryotes that are ubiquitous in virtually all environments. Many are grazers that feed by ingesting other microorganisms, especially bacteria. Protozoa have been interacting with bacteria for a very long time, and several species have become hosts of pathogenic bacteria in natural environments and in man-made structures (air conditioning units, cooling towers, etc.). Studying these interactions is particularly important given that protozoa, for example amoebae, can serve as natural reservoirs for bacteria such as *Legionella pneumophila* and *Mycobacterium* spp. (Greub and Raoult, 2004). This represents a health risk since these bacteria can be dispersed into the air when aerosols are produced and can cause severe, even lethal, pneumonia when inhaled (Abu Kwaik et al., 1998; Falkinham, 2003; Philippe et al., 2006). *L. pneumophila* can be propagated over long distances (several kilometers) while remaining infectious (Nguyen et al., 2006; Nygard et al., 2008). In addition to *L. pneumophila* and *Mycobacterium* spp., a large number of bacterial species can withstand predation by protozoa and can persist and/or grow in them. A summary of the outcomes reported in the literature for pathogenic bacteria that interact with various protozoa is presented in Table 1. The interactions of pathogenic bacteria with protozoa can be advantageous if they can resist predation and digestion by the protozoa. For example, *L. pneumophila* has developed a clever strategy to protect itself from the enzymatic degradation that normally occurs in the endocytic pathway of the host by inducing the formation of replication vacuoles inside protozoa (Richards et al., 2013). By being able to grow and survive inside protozoa, these resistant bacteria are protected from stresses like biocides and antibiotics.

**Table 1:** **Fate of bacteria following interactions with protozoa**.

**Protozoa**	**Fate of bacteria**	**References**
*Acanthamoeba astronyxis*	Intracellular survival^a^	Inglis et al., 2000; Marciano-Cabral and Cabral, 2003
	Intracellular multiplication	Marciano-Cabral and Cabral, 2003
	Packaged in expelled vesicles	Marciano-Cabral and Cabral, 2003
	Survival in cysts	Marciano-Cabral and Cabral, 2003
*Acanthamoeba castellanii*	Intracellular multiplication	Ly and Muller, 1990b; Cirillo et al., 1997; Essig et al., 1997; Winiecka-Krusnell et al., 2002; Abd et al., 2003; Casson et al., 2008; El-Etr et al., 2009; Saeed et al., 2009; Abd et al., 2010; Verhoeven et al., 2010; Lienard et al., 2011
	Intracellular survival^a^	King et al., 1988; Essig et al., 1997; Inglis et al., 2000; La Scola and Raoult, 2001; Winiecka-Krusnell et al., 2002; Abd et al., 2003, 2010; Thomas et al., 2006; Casson et al., 2008; El-Etr et al., 2009; Saeed et al., 2009
	Survival in cysts	Abd et al., 2003, 2010; El-Etr et al., 2009; Saeed et al., 2009; Cateau et al., 2011
	Long-term survival in vegetative forms or dead cells	Abd et al., 2003; Bouyer et al., 2007; El-Etr et al., 2009; Cateau et al., 2011
	Protection from chlorine	King et al., 1988
	Bacteria spore-like formation	La Scola and Raoult, 2001
	Biofilm formation	Verhoeven et al., 2010
	Protection from gentamicin	Bouyer et al., 2007; Saeed et al., 2009; Abd et al., 2010
	Packaged in expelled vesicles	Rowbotham, 1980; Berk et al., 1998
	Protection from biocides	Berk et al., 1998
	Protection from freezing and thawing	Berk et al., 1998
	Enhanced virulence	Cirillo et al., 1997
*Acanthamoeba comandoni*	Intracellular multiplication	Lienard et al., 2011
*Acanthamoeba culbertsoni*	Long-term survival in vegetative form	Cateau et al., 2011
	Survival in cysts	Cateau et al., 2011
*Acanthamoeba polyphaga*	Intracellular multiplication	Kilvington and Price, 1990; Barker et al., 1999; La Scola and Raoult, 1999; Birtles et al., 2000; Ingham et al., 2000; Kahane et al., 2001; La Scola et al., 2004; Evstigneeva et al., 2009; Ben Salah and Drancourt, 2010; Lamrabet et al., 2012
	Intracellular survival^a^	Steinert et al., 1998; Barker et al., 1999; Horn et al., 1999; Ingham et al., 2000; Inglis et al., 2000; La Scola et al., 2000, 2001, 2002, 2003, 2004; Kahane et al., 2001; Adekambi et al., 2006; Medie et al., 2011; Lamrabet et al., 2012; Lamrabet and Drancourt, 2013
	Survival in cysts	Kilvington and Price, 1990; Steinert et al., 1998; Horn et al., 1999; La Scola and Raoult, 1999; Kahane et al., 2001; Adekambi et al., 2006; Ben Salah and Drancourt, 2010; Medie et al., 2011
	Packaged in expelled vesicles	Berk et al., 1998
	Protection from chlorine	Kilvington and Price, 1990; La Scola and Raoult, 1999; Adekambi et al., 2006
	Protection from biocides	Berk et al., 1998
	Protection from freezing and thawing	Berk et al., 1998
	Protection from streptomycin and glutaraldehyde	Medie et al., 2011
*Acanthamoeba* spp.	Intracellular multiplication	Michel and Hauroder, 1997; Tomov et al., 1999
	Intracellular survival^a^	Drozanski, 1956; Ly and Muller, 1990a; Drozanski, 1991; Amann et al., 1997; Fritsche et al., 1999; Marolda et al., 1999; Tomov et al., 1999; Horn et al., 2001, 2002
	Survival in cysts	Horn et al., 2001, 2002
*Colpoda* spp.	Packaged in expelled vesicles	Raghu Nadhanan and Thomas, 2014
	Protection from gentamicin and chlorine	Raghu Nadhanan and Thomas, 2014
*Dictyostelium discoideum*	Intracellular multiplication	Hagele et al., 2000; Solomon and Isberg, 2000; Skriwan et al., 2002; Solomon et al., 2003; Hagedorn and Soldati, 2007; Hagedorn et al., 2009; Lienard et al., 2011
*Glaucoma* spp.	Intracellular multiplication	Gourabathini et al., 2008
	Packaged in expelled vesicles	Gourabathini et al., 2008
*Hartmannella vermiformis*	Intracellular multiplication	Lienard et al., 2011
	Intracellular survival^a^	Horn et al., 2000
*Naegleria gruberi*	Intracellular multiplication	Thom et al., 1992
	Survival in cysts	Thom et al., 1992
*Naegleria lovaniensis*	Intracellular multiplication	Casson et al., 2008
	Intracellular survival^a^	Casson et al., 2008
*Saccamoeba* spp.	Intracellular multiplication	Michel et al., 1995
*Tetrahymena pyriformis*	Intracellular multiplication	King and Shotts, 1988; Ly and Muller, 1990a,b; Gourabathini et al., 2008
	Intracellular survival^a^	King et al., 1988; Ly and Muller, 1990a
	Protection from chlorine	King and Shotts, 1988; King et al., 1988
	Packaged in expelled vesicles	Gourabathini et al., 2008
*Tetrahymena tropicalis*	Packaged in expelled vesicles	Berk et al., 2008; Koubar et al., 2011
	Long-term survival in vegetative form	Koubar et al., 2011
	Protection from gentamicin	Koubar et al., 2011
	Enhanced virulence	Koubar et al., 2011
*Tetrahymena* sp.	Intracellular survival	Smith et al., 2012
	Packaged in expelled vesicles	Brandl et al., 2005; Smith et al., 2012
	Long-term survival in vegetative form	Brandl et al., 2005
	Protection from low concentrations of calcium hypochlorite	Brandl et al., 2005

Positive outcomes for bacteria resulting from the interactions with protozoa are listed.

a In vacuoles or the cytoplasm.

Some investigators have suggested that protozoa may act as a Trojan Horse in the propagation of human pathogenic bacteria (Barker and Brown, 1994; Greub and Raoult, 2004). For example, soon after *L. pneumophila* was discovered, it was suggested that bacteria residing inside amoebae, rather than free bacteria, were the source of legionellosis (Rowbotham, 1980). *Legionella* and mycobacteria associated with *Acanthamoeba* have a greater capability to replicate in macrophages than free bacteria (Cianciotto and Fields, 1992; Cirillo et al., 1994, 1997, 1999; Moffat et al., 1994; Neumeister et al., 2000). *L. pneumophila*, *Mycobacterium* spp., and other amoeba-resisting bacteria may also be able to reside within amoebal cysts (Steinert et al., 1998; Marciano-Cabral and Cabral, 2003; Adekambi et al., 2006; El-Etr et al., 2009; Ben Salah and Drancourt, 2010). Most protozoa can make cysts, often with thick protective walls, which are their resting form, that provide protection from adverse environmental conditions (Greub and Raoult, 2004) and, at the same time, that provide involuntary protection to bacteria inside the cysts. *Mycobacterium* spp. that reside inside cysts can resist 15 mg/L of free chlorine for 24 h (Adekambi et al., 2006). Protozoal cysts may thus be vectors for some bacteria (Ben Salah and Drancourt, 2010).

## Bacteria packaging by protozoa: a bacterial camouflage

A broad range of phagotrophic protists such as dinoflagellates, ciliates, and amoebae produce and expel vesicles as a part of their normal digestive process (Gezelius, 1959; Hohl, 1965; Allen and Wolf, 1974, 1979; Buck et al., 1990, 2005; Buck and Newton, 1995; Chekabab et al., 2012; Paquet et al., 2013). These expelled vesicles, which are often called fecal pellets or fecal balls, vary in composition, size, and morphology, depending on the protist and the trophic conditions. These pellets, which contain, among things, undigested particulates, and organic nutrients, may play significant roles in the flux of materials in the ecosphere (Buck et al., 1990).

**Figure 1:**
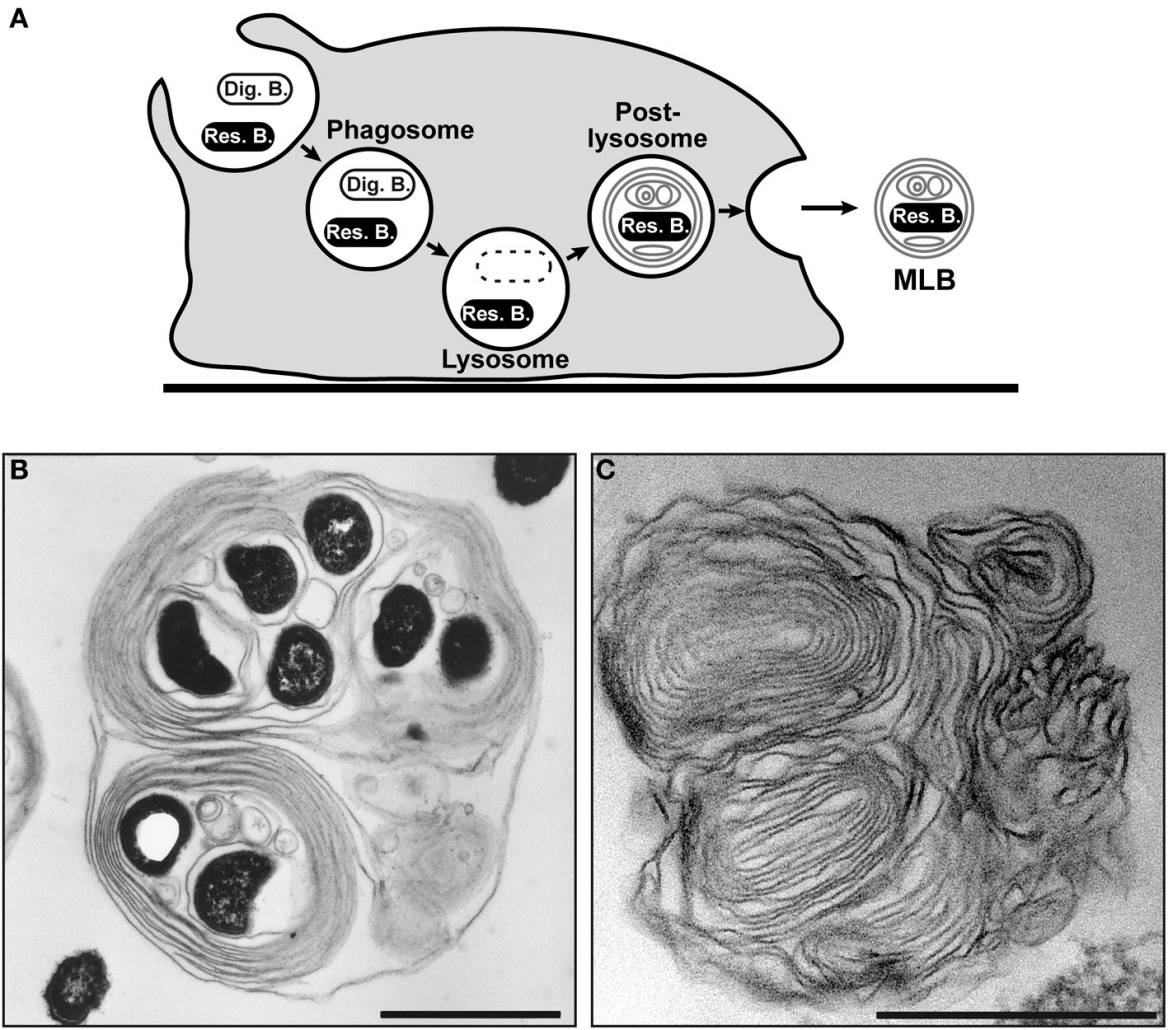
**Bacteria packaging by amoebae. (A)** Schematic diagram of the packaging process that allows packaged bacteria to resist lysosomal degradation (Res. B), unlike digestible bacteria (Dig. B). The resisting bacteria are packaged in multilamellar bodies (MLB) and are then secreted by the amoebae. **(B)** Transmission electron microscopic image of *L. pneumophila* bacteria (black ovoid forms) packaged in a MLB produced and secreted by *A. castellanii*. Image reproduced from Berk et al. (1998) with the permission of the American Society for Microbiology. **(C)** Transmission electron microscopic image of a MLB devoid of bacteria produced and secreted by *D. discoideum* DH1-10 (Cornillon et al., 2000) grown on digestible bacteria, which were a laboratory strain of *K. aerogenes* (Benghezal et al., 2006). Scale bar = 1 μm in **(B,C)**.

In addition, the long co-evolution of protists and bacterial preys has given rise to survival strategies by the bacteria that enable them to avoid digestion in the normal phagocytic process and to be packaged in the egested pellets. *Acanthamoeba* and *Tetrahymena* protozoa parasitized by *L. pneumophila* expel vesicles or fecal pellets that contain viable bacteria (Figure 1) (Rowbotham, 1980; Berk et al., 1998, 2008; Koubar et al., 2011). While bacteria packaging was first observed with *L. pneumophila*, it might be a general phenomenon since a variety of protozoa that expel various species of bacteria packaged in vesicles have been reported (Table 2). Amoebae and ciliates appear to be the only two groups of protozoa known to produce extracellular vesicles containing bacteria so far (Table 2). The vesicles are produced in the endocytic pathway either on a regular basis or under certain specific conditions (see below). Bacteria that resist lysosomal digestion can be packaged in these structures and can then be expelled outside the cell by exocytosis or, in some cases, following bacteria-dependent cell lysis (Figure 1) (Abd et al., 2003; Brandl et al., 2005; Gourabathini et al., 2008).

**Table 2:** **List of bacteria-protozoa combinations where bacteria packaging has been observed**.

**Bacterium**	**Protozoa**	**Output**	**References**
*Escherichia coli* O157	*Glaucoma* spp.	Packaged in expelled vesicles, multiplication, and escape from the vesicles	Gourabathini et al., 2008
	*Tetrahymena pyriformis*		
	*Tetrahymena* sp.	Intracellular survival, packaged in expelled vesicles	Smith et al., 2012
*Helicobacter pylori*	*Acanthamoeba astronyxis*	Packaged in expelled vesicles, intracellular multiplication	Marciano-Cabral and Cabral, 2003
*Legionella pneumophila*	*Acanthamoeba castellanii*	Packaged in expelled vesicles, resistance to biocides and freezing and thawing	Berk et al., 1998
	*Acanthamoeba polyphaga*		
	*Tetrahymena tropicalis*	Packaged in expelled vesicles	Berk et al., 2008
		Packaged in expelled vesicles, long-term survival, gentamicin resistance, increased infectivity	Koubar et al., 2011
*Listeria monocytogenes*	*Glaucoma* spp.	Packaged in expelled vesicles	Gourabathini et al., 2008
	*Colpoda* spp.	Packaged in expelled vesicles, resistance to sodium hypochlorite, gentamicin resistance	Raghu Nadhanan and Thomas, 2014
*Salmonella enterica*	*Glaucoma* sp.	Packaged in expelled vesicles	Gourabathini et al., 2008
	*Tetrahymena pyriformis*		
	*Tetrahymena* sp.	Packaged in expelled vesicles, enhanced survival, resistance to low concentrations of calcium hypochlorite	Brandl et al., 2005

Packaged bacteria are protected against harsh conditions such as freezing and thawing, chlorine, and the biocides used in cooling towers (Berk et al., 1998; Brandl et al., 2005). *Tetrahymena tropicalis*-packaged *L. pneumophila* display greater gentamicin resistance and long-term survival in nutrient-poor environments, and are more infectious than free bacteria in cultured human pneumocyte cells (Koubar et al., 2011). More recently, it has been reported that viable *Listeria monocytogenes* can be enclosed in fecal pellets produced by *Colpoda* spp. (a ciliate) and that this results in gentamicin and sodium hypochlorite resistance of the bacteria (Raghu Nadhanan and Thomas, 2014). Packaging may thus be a way for the bacteria to persist in the environment. In fact, bacteria packaged by protozoa are more likely to propagate bacterial infections than bacteria-containing cysts. Packaged bacteria are probably the most frequent form of pathogenic bacteria associated with protozoa. Exocytosis is a continuous active process for grazing protozoa and hundreds of bacteria-containing vesicles can be expelled by a single protozoal cell (Berk et al., 1998; Brandl et al., 2005; Gourabathini et al., 2008). However, protozoal cells can form only a single bacteria-containing cyst and while bacteria have been observed in protozoal cysts, they are not always viable in these structures (Gourabathini et al., 2008).

Packages containing bacteria range in size from 2 to 6 μm in diameter (Berk et al., 1998, 2008) and are smaller than vegetative forms of protozoa and even cysts, which can reach diameters of 10–20 μm (Nilsson, 2005). Given that respirable particles (i.e., those able to penetrate into the alveoli) must be under 3.5 μm in diameter (Macher, 1999), packaged bacteria can thus penetrate deeper into the respiratory tract and the alveoli. Respiratory pathogens such as *L. pneumophila* that can be packaged are thus more likely to cause respiratory infections in this form than when included in vegetative forms of protozoa or cysts (Berk et al., 1998). It has been proposed that bacteria that develop inside amoebae may be responsible for some of the 50% of lower respiratory tract infections with unexplained etiologies (Lamoth and Greub, 2009). While the packaging process needs to be studied in greater detail, it cannot be excluded that packaged bacteria may cause some of these lower respiratory tract infections.

## Bacteria packaging: a protozoa-driven process

The packages produced by amoebae (Figure 1B) and, to a lesser extent by ciliates (see below), are multilamellar bodies (MLBs) formed of several concentric layers of lipid membranes containing viable bacteria. MLBs devoid of viable bacteria are also produced (Figure 1C), for example by *Acanthamoeba castellanii* (Chekabab et al., 2012), and have been extensively studied using *Dictyostelium discoideum* amoeba (Gezelius, 1959; Mercer and Shaffer, 1960; Gezelius, 1961; Hohl, 1965; Barondes et al., 1985; Cooper et al., 1986; Fukuzawa and Ochiai, 1993; Emslie et al., 1998; Marchetti et al., 2004; Paquet et al., 2013).

To date, the production of MLBs by *D. discoideum* has only been studied in the presence of digestible bacteria (i.e., bacteria that are degraded by the lysosomal enzymes of the endocytic pathway) and not in the presence of undigestible pathogenic bacteria. Interestingly, since *D. discoideum* cells grown in liquid medium in the absence of bacteria produce virtually no MLB (Mercer and Shaffer, 1960; Hohl, 1965; Marchetti et al., 2004; Paquet et al., 2013), it was considered that these MLBs were undigested bacterial remains. However, new evidence has shown that MLB production by *D. discoideum* depends largely on the metabolism of the protozoa even if the presence of digestible bacteria is required to produce MLBs. The analysis of the composition of purified *D. discoideum* MLBs revealed that the lipids in these structures are amoebal in origin, that is, they are mainly produced via amoebal metabolic pathways, even if they are only produced when the amoebae are fed *Klebsiella aerogenes*, a digestible bacterium (Paquet et al., 2013). Even if bacterial remains (glycoconjugates) have also been detected in *D. discoideum* MLBs (Cooper et al., 1986), the lipid composition of these structures suggests that they are not strictly fecal pellets used to dispose of undigested bacterial constituents and that they may play significant roles in amoebal physiology.

**Figure 2:**
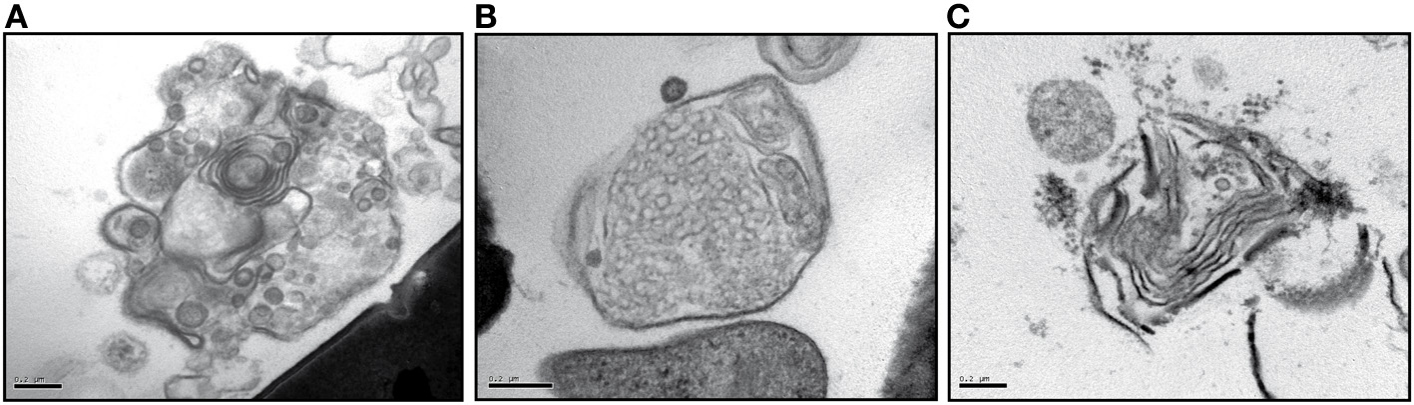
**MLBs secreted by *D. discoideum* cells grown on Gram-positive bacteria**. Transmission electron microscopic images of MLBs secreted by *D. discoideum* DH1-10 cells (Cornillon et al., 2000) grown on *B. subtilis* (Benghezal et al., 2006) **(A,B)** and *M. luteus* ATCC 4698 **(C)**. One MLB is shown in each panel. Scale bars = 0.2 μm.

The fact that the lipids in the MLBs are amoebal in origin is an argument in favor of the idea that bacteria packaging is under the control of protozoa. However, when *D. discoideum* cells are fed digestible Gram-positive bacteria (Figure 2) compared to digestible Gram-negative bacteria (Figure 1), the type of food, that is, the type of bacteria ingested, can affect the morphology of the MLBs. Those produced and secreted by *D. discoideum* cells grown on *Bacillus subtilis* and *Micrococcus luteus* (Gram-positive bacteria) are quite different than those from amoebae grown on *K. aerogenes* (Gram-negative bacterium). In the presence of *B. subtilis*, the MLBs have a more heterogeneous structure (Figures 2A,B) that can be composed of concentric membrane lamellae, clusters of vesicles in one structure, or a mix of the two. MLBs produced and secreted by cells grown on *M. luteus* contain fewer lamellae and often have a broken appearance (Figure 2C). Without being the main actor in bacteria packaging, internalized bacteria may produce factors that influence the process. For example, *L. pneumophila* LepA and LepB proteins appear to be essential for the specific non-lytic release of the bacteria from amoebae (Chen et al., 2004). Do these proteins act as a kind of regulator of the packaging process or only of the exocytosis function? Additional studies are required to evaluate the contribution of bacterial factors to the packaging process.

Less is known about the nature of the packages produced by ciliates than those produced by amoebae. The composition and structure of the packages or pellets produced by ciliates such as *Tetrahymena* spp. differ from those of amoebal MLBs. Packaged bacteria secreted by *Tetrahymena* spp. appear to have various profiles as shown by a transmission electron microscopy study of pellets containing *L. pneumophila* (Berk et al., 2008). The pellets had three morphologies: (1) bacteria embedded in abundant membranous and vesicular material reminiscent of amoebal MLBs, (2) bacteria surrounded by amorphous material, and (3) bacteria with no apparent electron dense material surrounding them. *Tetrahymena* spp. have also been induced to package *Escherichia coli* O157:H7 (Smith et al., 2012). Scanning electron microscopy has revealed that the pellets produced in this case have a net-like structure surrounding the bacteria and aggregate in flocs due to their stickiness. This suggests that packages produced by ciliates can vary in form and composition as with amoebae. However, it is difficult to determine the extent of the variability without an exhaustive side-by-side comparison of packages produced by various protozoa packaging the same bacterial species.

Like amoeba, ciliates are able to produce MLBs when fed digestible bacteria (Berk et al., 2008). These MLBs are identical to those produced by *D. discoideum* fed digestible bacteria. This suggests that ciliates also control the production of pellets. Immunolabeling has shown that the membranous material in packages produced by *Tetrahymena* spp. is, in part, of bacterial origin (Berk et al., 2008), and indicates that pellets produced by ciliates are likely a mosaic composed of protozoal and bacterial material similar to MLBs produced by amoebae.

**Figure 3:**
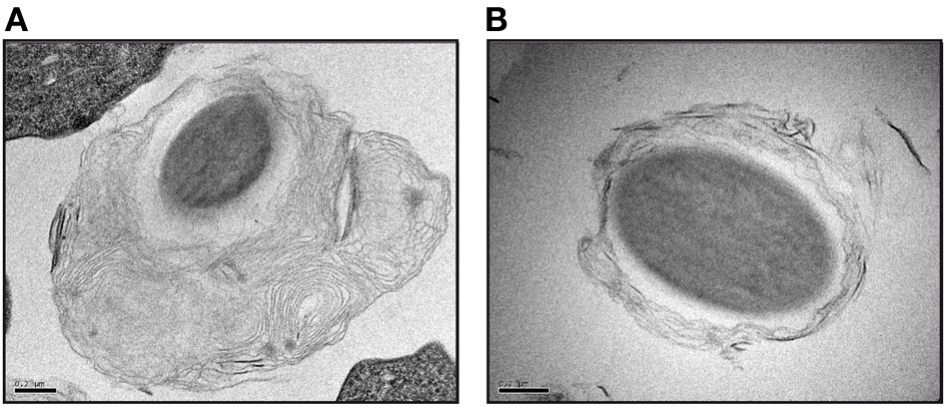
***D. discoideum* cells can package polystyrene beads in secreted MLBs**. Transmission electronic micrographic images of polystyrene beads packaged in thick **(A)** and thin **(B)** MLBs after being incubated with *D. discoideum* DH1-10 cells (Cornillon et al., 2000) in the presence of digestible bacteria. Scale bars = 0.2 μm.

The most convincing proof that bacteria packaging is a protozoa-driven process comes from experiments where protozoa are fed synthetic beads. Since the primary characteristic of pathogenic bacterial parasites of protozoa is resistance to enzymatic degradation in phago-lysosomes (Molmeret et al., 2005), the synthetic beads mimic undigestible bacteria but are inert and do not biochemically interact with the protozoa. When fed latex beads, *Tetrahymena* cells produced packaged beads in a net-like matrix similar to that observed with *E. coli* (Smith et al., 2012). Similarly, *D. discoideum* cells grown in presence of polystyrene beads and digestible bacteria produced MLBs containing beads (Figure 3). These results are of the utmost important since they indicate that the packaging process is independent of the ingested particle and is mainly under the control of the protozoa as long as the production of packages is metabolically stimulated.

## Hypothesis and perspectives

Since packages can be produced in abundance by protozoa and provide bacteria enclosed in the structures with much greater resistance to unfavorable conditions, it is tempting to hypothesize that bacteria packaging by protozoa is a general process that contributes to the survival and propagation of pathogenic bacteria in the environment. This process may be an unsuspected source of pathogenic bacteria that could explain many infections, including some of the respiratory tract. The conditions that favor the production of packaged bacteria and their distribution in natural environments and man-made structures are unknown. It will be necessary to identify the environments and conditions in which packaged bacteria are produced in the real world. This cannot be achieved without conducting field studies to first check for the presence of protozoa harboring intracellular bacteria and then to quantitatively assess the presence of packaged bacteria. This information will help in the development of strategies to prevent the spread of pathogenic bacteria. To confirm this hypothesis, many elements need to be addressed.

**Figure 4:**
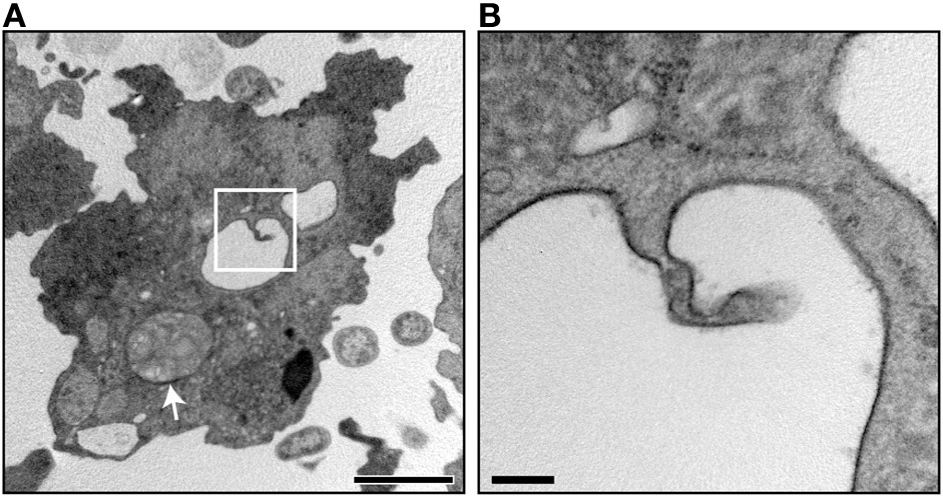
**Intra-lysosomal profile of *D. discoideum* cells fed digestible bacteria. (A)** Transmission electronic micrographic image of a *D. discoideum* DH1-10 cell (Cornillon et al., 2000) with a lysosomal compartment displaying an intra-lysosomal profile (white square) and a MLB inside a lysosomal compartment (white arrow). B. Magnification of image A showing the inward bud in greater detail. The inward budding is in a lysosomal compartment containing no other electron dense material. The invaginations of the lysosomal membrane are hard to detect in compartments already containing MLBs because the compartments are too crowded. Scale bar = 2 μm in **(A)** and 0.2 μm in **(B)**.

First, it could be interesting to determine whether other players are involved in bacteria packaging. Even if many protozoa and bacteria are known to participate in this process (Table 2), it would be interesting to determine how many other pathogenic and even non-pathogenic bacteria can be packaged as well as which protozoa can perform the packaging. This may be quite difficult since some environments in which bacteria packaging may occur cannot be easily reproduced *in vitro*. In addition, some bacterial species may be packaged by one type of protozoa but not by another. Bacteria packaging may also occur only in presence of a tripartite interaction as with *D. discoideum* where digestible bacteria are required to produce MLBs containing synthetic beads (Figure 4) and with *A*. *castellanii*, where the production of packaged *L. pneumophila* is enhanced in the presence of digestible *E. coli* (Berk et al., 1998). Environmental conditions likely play a role in the yield of packaged bacteria and even in the process itself. For example, the resistance of *L. pneumophila* to *Acanthamoeba palestinensis* predation can be modulated by environmental conditions such as the incubation temperature (Anand et al., 1983), which may affect the capacity of the bacteria to be included in packages.

Nothing is known about the protozoal mechanisms involved in bacteria packaging since research to date has mainly focused on the impacts of packaging on the bacteria. Apart from the advantages packaging provides to bacteria (Table 2), research has also focused on the transcriptional response of *Salmonella enterica* during packaging and the requirement of *L. pneumophila* for a functional Dot/Icm system to resist protozoal degradation and be packaged (Berk et al., 2008; Rehfuss et al., 2011). However, given that the packaging process is under the control of protozoa, a better understanding of the molecular mechanisms of the protozoal endocytic pathway involved in the packaging process is required.

MLB production by *D. discoideum* cells grown in liquid culture can be stimulated by U18666A, a drug that disrupts intracellular cholesterol transport and metabolism in mammalian cells. Under these conditions, MLBs are produced by the invagination of the membrane inside the lysosomal compartments (Marchetti et al., 2004). Inward budding also occurs when *D. discoideum* cells are fed digestible bacteria (Figure 4), suggesting that lysosomal membrane proteins may be included in MLBs. In fact, amoebal proteins such cysteine proteinase and discoidin I as well as other unidentified glycosylated proteins have been already detected in secreted MLBs (Barondes et al., 1985; Fukuzawa and Ochiai, 1993; Emslie et al., 1998; Paquet et al., 2013).

The identification of all the protozoal proteins in the MLBs used to package bacteria as well as the biological machinery involved in the process and their characterization will open up new avenues for understanding the packaging process. For example, the identification of the proteins included in MLBs will provide cues to the packaging mechanisms as well as markers for visualizing the packaging process by real-time microscopy. The identification of these proteins and mechanisms will make it possible to develop tools that will help to address important research issues related to bacteria packaging by protozoa such as their presence in the environment and their role in infectious diseases. Understanding the mechanisms should also make it possible to develop chemical inhibitors or modulators of bacteria packaging.

In addition to identifying protozoal proteins in the packages, another approach to understand the mechanisms involved in the process would be to identify the proteins that are essential for the packaging process. Since the production of MLBs is similar to the production of multivesicular bodies (MVBs) by many eukaryotic organisms (Piper and Katzmann, 2007), it is highly likely that the ESCRT complexes involved in MVB biogenesis are also involved in the production of packaged bacteria. Among others, autophagy is probably also associated with MLB production since a link exists between autophagy and MVB biogenesis (Fader and Colombo, 2009).

The *D. discoideum* model appears to be promising for the identification of the genes encoding the proteins involved in the packaging process. Site-directed mutagenesis is a routine procedure with *D. discoideum*, and a mutant of *tom1*, which encodes one of the proteins of an ESCRT-like complex in this amoeba, has already been developed (Blanc et al., 2009). Mutants of the gene encoding Alix, a protein functionally associated with ESCRT complexes, as well as of genes encoding proteins involved in autophagy are also already available (Mattei et al., 2006; Calvo-Garrido et al., 2010). Studying the capacity of these mutants and others that can be generated in the future to produce normal MLBs appears to be a good approach for shedding light on the mechanisms involved in bacteria packaging.

In addition to studying the mechanisms of package formation by protozoa, the impact of packaged bacteria on human health must be investigated. The most probable assumption is that packaged bacteria can be aerosolized, spread over long distances, and play a role in the transmission of respiratory tract infections. Since aerosolization is a major route of transmission of many human pathogens and is also a significant stress factor for microorganisms (Macher, 1999), it is important to determine the relative viability of packaged bacteria compared to free bacteria following aerosolization. It is also important to determine the response of aerosolized packaged bacteria to environmental stresses such as ultraviolet radiation (UV).

Previous studies have provided support for the idea that the intracellular growth of pathogenic bacteria in protozoal hosts increases the invasive potential and virulence of the bacteria in mammals (Cirillo et al., 1994, 1997, 1999; Molmeret et al., 2005). By producing packaged bacteria, protozoa may help bacteria to remain undetected by the immune system following inhalation, which in turn may help them to better adapt to their new environment like the respiratory tract and be more effective in the development of an infection. While the role of bacteria packaging in the infectious process has never been clearly addressed, *T. tropicalis*-packaged *L. pneumophila* are much more infectious than free bacteria in cultured human pneumocyte cells (Koubar et al., 2011). Are packaged bacteria more infectious than free bacteria in animal models? How does the immune system response to packaged and free bacteria differ? Answers to these questions are fundamental because protozoa can produce hundreds of packages containing pathogenic bacteria that can persist even after the protozoa have disappeared.

## Conclusion

Research to date has been limited to descriptions of bacteria packaging by various protozoa. A number of findings suggest that packaging provides the bacteria with an advantage in the environment and can contribute to their pathogenicity. However, many elements need to be clarified to determine whether packaged bacteria are a significant source of infections.

It will be essential to extend the characterization of the role played by bacteria packaging to the persistence of pathogens in the environment and their ability to cause infections, especially of the respiratory tract. More precisely, we require a better understanding of the mechanisms involved in bacteria packaging, the magnitude of this phenomenon in various environments, the enhanced virulence potential related to the increased propagation of bacteria, and their ability to cause infections. The development of packaging markers, protocols for producing packaged bacteria, and methods to detect these bacteria are also needed to better understand this process. Ultimately, if bacteria packaging appears to make an important contribution to the persistence and transmission of pathogenic bacteria, it will be possible to reduce their infectivity and propagation by modulating their interactions with protozoa.

### Conflict of interest statement

The authors declare that the research was conducted in the absence of any commercial or financial relationships that could be construed as a potential conflict of interest.
